# Source analysis and ecological risk assessment of potentially toxic elements in farmland soil around a mining area

**DOI:** 10.1371/journal.pone.0351832

**Published:** 2026-06-17

**Authors:** Ying Hu, Jiantong Lu, Yan Gao, Xiaotang Ye, Chi Zhang, Jiankuan Li, Hewan Huang, Chenxi Li

**Affiliations:** Second Geological Brigade of Shandong Geological and Mineral Exploration and Development Bureau, Shandong Lunan Geological Engineering Survey Institute, Jining, China; Universidade de Vigo, SPAIN

## Abstract

In the context of the interplay between ecology, society, and economy, analyzing the characteristics of soil heavy metal pollution and constructing ecological security patterns have emerged as critical scientific issues that necessitate urgent attention for the advancement of high-quality agricultural development. This study focused on the farmland surrounding the Baiyun Mountain mining area in Nanyang, China, and involved the collection of 437 surface soil samples (0–20 cm) to analyze the spatial differentiation characteristics of nine heavy metal elements, including Ag, Cu, and Pb. An ecological risk assessment model was developed to evaluate the ecological risks posed by potentially toxic elements in the region, based on the theory of matter element extension. Additionally, the Positive Matrix Factorization (PMF) model was employed to analyze the sources of heavy metal pollutants. The results indicated that: (1) The average concentrations of Ag, Cu, Pb, Zn, Mo, Sb, Ba, As, and Hg were 0.06, 17.50, 22.72, 66.84, 1.15, 0.85, 426.39, 9.25, and 0.05 mg·kg^-1^, respectively, with Zn, Mo, and Hg significantly exceeding the soil background values for Henan Province; (2) The ecological risk assessment of potentially toxic elements revealed that 93.82% of the samples were classified as clean, 5.49% as at mild risk, and 0.69% as at severe risk, with Hg identified as the primary ecological risk factor; (3) The primary sources of pollution can be categorized as follows: natural parent material sources (26.1%), mixed sources from transportation and mining development (25.8%), mixed sources resulting from pesticide use and mining emissions (19.8%), atmospheric deposition from fossil fuel combustion (15.1%), and sources from non-metallic mineral mining and building material industry (13.2%). This study provides a scientific basis for the prevention and control of soil pollution in farmland located in mining areas.

## 1. Introduction

Soil, as a vital natural resource that underpins human survival and global food security, not only facilitates various production activities but also serves as the core foundation of the agricultural production system within the “One Health” framework [1–3]. However, the acceleration of global industrialization and the intensive development of mineral resources have not only stimulated regional economic growth but have also led to complex pollution issues at the soil-water-atmosphere interface surrounding mining areas [4]. Among these issues, heavy metal pollutants present a significant challenge in the realm of global soil ecological remediation due to their persistence, concealment, and bioaccumulation characteristics [5]. Heavy metals in agricultural land not only diminish crop yields but also accumulate through the food chain, posing a long-term threat to ecosystem stability and human health [6]. Therefore, analyzing the sources of heavy metal pollution and the ecological risks within the mining-agricultural complex system holds considerable academic value and practical significance for facilitating precise prevention and ecological restoration of contaminated farmland.

Traditional ecological risk assessments of heavy metal pollutants often rely on methods such as the Nemerow comprehensive pollution index, geological cumulative index, ecological hazard index, and fuzzy comprehensive evaluation [7–9]. However, the Nemerow index method struggles to quantify the nonlinear interactions between pollutants [10,11]. The geological accumulation index method lacks a systematic assessment of the bioavailability of potentially toxic elements, leading to discrepancies between ecological risk assessment results and actual exposure scenarios. Furthermore, the fuzzy comprehensive evaluation method may overlook significant information represented by small measurement values during the synthesis of fuzzy operators, resulting in substantial errors in evaluation outcomes [12,13]. In contrast, the matter element extension model constructs a three-dimensional evaluation system of ‘concentration-toxicity- occurrence mode’ through dynamic extension set theory, which can quantitatively characterize the synergistic and antagonistic effects between potentially toxic elements [14,15]. Compared to traditional pollution index methods, the evaluation results from this model are highly reliable and objective, and it has been widely used to assess the ecological risk issues of various pollutants [16,17]. Therefore, this study introduces the theory of matter element extension to construct a multidimensional matter element matrix for the quantitative assessment of the ecological risk of heavy metal pollution.

In the field of pollution source apportionment, receptor models have emerged as mainstream techniques due to their capacity for quantitative source tracking through the interpretation of the “chemical fingerprints” of environmental media. Common receptor models include the Chemical Mass Balance (CMB) model, the UNMIX model, and the Positive Matrix Factorization (PMF) model [18–20]. Although statistical methods such as Principal Component Analysis (PCA) are widely employed, they often struggle to address the uncertainties inherent in environmental measurement data. The CMB model, which relies heavily on established source composition profiles, has limited applicability in complex mining areas characterized by multiple intertwined sources [21]. In contrast, the PMF model exhibits greater robustness by incorporating measurement errors and employing non-negative constraint algorithms, thereby providing analytically meaningful results without necessitating prior knowledge of source composition profiles. Recently, scholars both domestically and internationally have conducted a series of studies focusing on various regional characteristics. For instance, the homology migration patterns of Cu, Pb, and Zn have been elucidated through PMF-GIS coupling technology; research in agricultural areas of the Ganges Plain, India, indicates that fertilizer application contributes to over 40% of soil Cd and Hg accumulation [22,23]. By analyzing the multi-source input characteristics of Sb, As, and Hg in the antimony mining area of Hunan, China, the enduring impact of historical mining activities has been confirmed [24]. However, existing research has primarily focused on conventional elements such as Pb, Cd, and As, while insufficient attention has been given to potential toxic elements that are closely related to specific deposit types, such as Mo, Sb, Ba, and Ag [25–27]. This oversight may lead to interference among components in the receptor model when attempting to isolate independent contributions from mining activities due to overlapping source profiles. Ultimately, this can result in misjudgments of contribution rates and significant deviations in analytical results [28,29].

Although the assessment of toxic elements in farmland soils surrounding mining areas has garnered significant attention, the existing evaluation systems are often restricted to macroscopic descriptions of pollution levels. Furthermore, there remains an inadequate differentiation of the toxic effects among various elements, as well as a lack of deep correlation with specific mineralization geochemical characteristics. Additionally, the distribution patterns and source contributions of trace elements such as Mo, Sb, and Ag, which serve as significant mineralization indicators, still lack systematic differentiation. Therefore, this study focuses on the sources and ecological risks of potentially toxic elements in farmland soil. It investigates the farmland surrounding the Baiyun Mountain mining area in Nanyang, China, and soil heavy metal analysis data were obtained. The aims are to: (1) Analyze the spatial distribution patterns of nine potentially toxic elements, including Ag, Cu, Pb, Zn, Mo, Sb, Ba, As, and Hg; (2) Construct a soil heavy metal ecological risk assessment model based on the theory of matter element extension to quantitatively evaluate the ecological risk levels of potentially toxic elements in farmland soils around mining areas; (3) Utilize the PMF receptor model and Kriging spatial interpolation technique to quantitatively analyze the multiple mixed sources and contribution rates of potentially toxic elements in agricultural mining composite systems. This research provides a scientific basis for the precise prevention and control of soil pollution and the formulation of remediation strategies for farmland in mining areas.

## 2. Materials and methods

### 2.1. Overview of the research area

This study area is situated in the western part of Nanyang, China (113°43’ ~ 113°45’ E, 32°47’ ~ 32°49’ N), at the junction of the Funiu Mountains and the Tongbai Mountain Range. It is classified as a tectonic erosion low mountain and hilly landform unit. The overall terrain exhibits a descending trend from southwest to northeast, with an average altitude of 625 m. The diversity of landforms and the complexity of terrain variations significantly influence the migration of potential toxic elements in the region. The steep slopes and dense valleys are susceptible to leaching and accumulation of surface soil and mining debris, exacerbated by erosion from high-intensity summer rainfall, resulting in notable spatial variations in heavy metal concentrations across different slope positions. The region’s climate is characterized by a monsoon humid climate, transitioning from the northern subtropical zone to the warm temperate zone, with an annual average temperature of 15.1 °C and annual precipitation of 971 mm, of which 62% occurs from June to August. This pronounced seasonal precipitation further intensifies slope erosion. The predominant soil types in the study area are yellow-brown soil (63.2%), which has developed from the parent material of Xia Shu loess during the Quaternary period, and tidal soil (28.5%), derived from river and lake sediments [30]. The sedimentary landforms, shaped by the development of river valleys and alluvial deposits such as tidal soil, exhibit distinct material accumulation characteristics influenced by complex river dynamics. This dynamic process of sedimentation and geomorphic evolution further exacerbates the heterogeneity of heavy metal distribution within the region. The region is notably endowed with mineral resources, including abundant metal minerals such as Fe, Cu, and Pb-Zn, as well as non-metallic minerals like limestone, cement-grade limestone, and brick and tile shale.

### 2.2. Sample collection and testing

**Fig 1:**
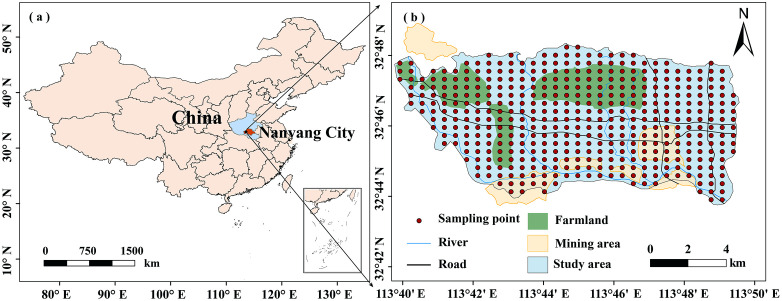
Overview of the study area and distribution of sampling sites. All map layers were created by the authors. Administrative boundaries were obtained from the National Catalogue Service for Geographic Information of China (http://www.webmap.cn/). Land-use data and infrastructure locations were digitized based on the authors’ field survey coordinates. No copyrighted basemaps were used in the production of these figures.

This study performed soil sampling in the farmland surrounding the Baiyun Mountain mining area in Nanyang, China in July 2023. The sampling design adhered to the principle of multi-level nesting. Primary grids were established at a density of 0.2 km^2^ (horizontal spacing of 500 ± 10 m, vertical spacing of 400 ± 10 m), while secondary units employed a five-point plum blossom-shaped sampling method. Specifically, a 5 m × 5 m sampling area was defined with the grid center as the origin. Samples were collected from the top vertices of the four quadrants (with coordinate offsets of ±0.5 m) and the central point at a depth of 0–20 cm within the cultivated layer. Sub-sample points were mixed in equal mass ratios (1:1) to form composite samples, resulting in a total of 437 effective soil samples (Fig 1). During the sampling process, the geographical coordinates of the sampling points were meticulously recorded. After sampling, the samples should be naturally dried in a dark and ventilated environment. Following air drying, any foreign objects such as gravel and plant debris are removed, and the samples are then crushed using a wooden stick. All samples are passed through a 2 mm nylon sieve to obtain a uniform fine soil sample. A portion of the sieved sample is weighed using the quartering method and subsequently ground with an agate mortar until all samples pass through a 150 μm nylon sieve. The sample preparation process strictly adheres to the “Technical Specifications for Soil Environmental Monitoring” (HJ/T 166–2004), ensuring the uniformity and detection accuracy of subsequent chemical digestion and total analysis of heavy metals. Throughout the operation, non-metallic tools were exclusively used to minimize the risk of introducing metallic contaminants.

The analysis of heavy metal elements in soil is conducted using a multi-instrumental combined technology to satisfy the detection requirements for various elements. Before instrument measurement, soil samples must be classified and digested according to the characteristics of the target elements. For the detection of arsenic (As), antimony (Sb), and mercury (Hg) elements, 0.5000 g of the soil sample was weighed and placed in a colorimetric tube. The samples were treated with and digested in aqua regia, which was prepared by mixing concentrated HCl and HNO_3_ in a volume ratio of 3:1. After cooling, the sample was adjusted to a constant volume for measurement using a BAFS-8520 atomic fluorescence spectrometer. The detection process for Hg follows the analytical scheme established in reference [31], while the analysis of As strictly adheres to the technical specifications outlined in “Determination of Total Mercury and Total Arsenic in Soil by Atomic Fluorescence Method Part 2: Determination of Total Arsenic in Soil” [32]. The quantification of Sb follows the technical specifications in “Determination of Antimony in Soil by Atomic Fluorescence Method” [33]. For elements such as lead (Pb), copper (Cu), silver (Ag), zinc (Zn), molybdenum (Mo), and barium (Ba), 0.1000 g of soil sample was weighed and placed in a polytetrafluoroethylene crucible. The HCl-HNO_3_-HF-HClO_4_ tetraacid electric heating plate digestion method was employed, and the sample was heated until it became dry and free of black residue. Subsequently, it was extracted with dilute nitric acid to a constant volume. The sample solutions were analyzed using a PinAAcle 900T atomic absorption spectrometer (for the determination of Cu, Zn, Pb, with reference to [34]) and an XSERIES2 inductively coupled plasma mass spectrometer (for the determination of Ag, Mo, Ba, and low concentration Pb). During the experimental process, all detection methods were rigorously validated, and quality control procedures were implemented for all analytical processes to ensure data reliability. The overall quality control results indicated that the recovery rate of each element ranged from 90% to 110%, and the relative standard deviation (RSD) of repeated sample determinations was less than 10%. These findings confirm that the data quality of this study is reliable and can support subsequent risk assessments and source analyses (S1 Table).

### 2.3. Matter-element extension model

(1) Constructing a matter element matrix for ecological risk assessment of potentially toxic elements

The matter-element matrix R is denoted as *R* = (*S*,*X*,*C*), where *S* is the object to be evaluated, *X* is the risk factor, and *C* is the quantity value. If the object *S* to be evaluated has *n* risk factors corresponding to *n* quantity values, it can be expressed as:


$$\begin{array}{c} R=\left(E,F_{n},V_{n}\right)=(\begin{array}{cc} \begin{array}{c} S \end{array} & \begin{array}{cc} X_{\text{1}} & C_{\text{1}}\\ X_{\text{2}} & C_{\text{2}}\\ \vdots & \vdots \end{array}\\ & \begin{array}{cc} X_{n} & C_{n} \end{array} \end{array}) \end{array}$$ (1)

(2) Determination of the classical domain and section domain

The matter-element matrices of the classical domain *R* (*j*) and the node domain *R* (*p*) of the ecological risk level of potentially toxic elements in soil are:


$$\begin{array}{c} R(j)=\left(S_{j},X_{i},C_{i}\right)=(\begin{array}{cc} \begin{array}{c} S_{j} \end{array} & \begin{array}{cc} X_{\text{1}} & C_{j\text{1}}\\ X_{\text{2}} & C_{j\text{2}}\\ \vdots & \vdots \end{array}\\ & \begin{array}{cc} X_{n} & C_{jn} \end{array} \end{array})=(\begin{array}{cc} \begin{array}{c} S_{j} \end{array} & \begin{array}{cc} X_{\text{1}} & \left(\alpha_{j\text{1},}\beta_{j\text{1}}\right)\\ X_{\text{2}} & \left(\alpha_{j\text{2},}\beta_{j\text{2}}\right)\\ \vdots & \vdots \end{array}\\ & \begin{array}{cc} X_{n} & \left(\alpha_{jn,}\beta_{jn}\right) \end{array} \end{array}) \end{array}$$ (2)

$$\begin{array}{c} R(p)=\left(S_{p},X_{i},C_{i}\right)=(\begin{array}{cc} \begin{array}{c} S_{p} \end{array} & \begin{array}{cc} X_{\text{1}} & C_{p\text{1}}\\ X_{\text{2}} & C_{p\text{2}}\\ \vdots & \vdots \end{array}\\ & \begin{array}{cc} X_{n} & C_{pn} \end{array} \end{array})=(\begin{array}{cc} \begin{array}{c} S_{p} \end{array} & \begin{array}{cc} X_{\text{1}} & \left(\alpha_{p\text{1},}\beta_{p\text{1}}\right)\\ X_{\text{2}} & \left(\alpha_{p\text{2},}\beta_{p\text{2}}\right)\\ \vdots & \vdots \end{array}\\ & \begin{array}{cc} X_{n} & \left(\alpha_{pn,}\beta_{pn}\right) \end{array} \end{array}) \end{array}$$ (3)

where (*α*_*jn*_*, β*_*jn*_) represents the range of values corresponding to level j for the risk factor Xn; (*α*_*pn*_*, β*_*pn*_) represents the value range of the element of the domain regarding the risk factor *X*_*n*_. Obviously, (*α*_*jn*_*, β*_*jn*_) is a subset of (*α*_*pn*_*, β*_*pn*_) (*n* = 1, 2, 3, …, *i*).

(3) Determination of the correlation function and calculation of the correlation degree *K*_*j*_(*C*_*i*_)

In the object-element theory system, the correlation function *K*_*j*_(*C*_*i*_) serves as a quantitative analysis tool to detect the degree of conformity with the standard level. When *K*_*j*_(*C*_*i*_) ∈ [0, +∞), it indicates that the evaluated object element complies with the standard; when *K*_*j*_(*C*_*i*_) ∈ (−1, 0], it means that the evaluated object element has not met the benchmark requirements but has the possibility of achieving compliance through regulatory means; when *K*_*j*_(*C*_*i*_) ∈ (- ∞ , −1], it indicates that the evaluated object element does not meet the standard. The function is as follows:


$$\begin{array}{c} K_{j}\left(C_{i}\right)=\left\{ \begin{array}{c} -\frac{\rho \left(C_{i},C_{ji}\right)}{\left|C_{ji}\right|}\;C_{i}\in C_{ji}\\ \frac{\rho (C_{i},C_{ji})}{\rho \left(C_{i},C_{Pi}\right)-\rho (C_{i},C_{ji})}\;{\;C}_{i}\notin C_{ji} \end{array}\right.\; \end{array}$$ (4)

$$\begin{array}{c} C_{ji}=|\beta_{jn}-\alpha_{jn}| \end{array}$$ (5)

$$\begin{array}{c} \rho \left(C_{i},C_{ji}\right)=\left|C_{i}-\left(\beta_{jn}+\alpha_{jn}\right)\times \text{0}.\text{5}\right|-(\beta_{jn}-\alpha_{jn})\times \text{0}.\text{5} \end{array}$$ (6)

$$\begin{array}{c} \rho \left(C_{i},C_{pi}\right)=\left|C_{i}-\left(\beta_{pn}+\alpha_{pn}\right)\times \text{0}.\text{5}\right|-(\beta_{pn}-\alpha_{pn})\times \text{0}.\text{5} \end{array}$$ (7)

where *ρ*(*C*_*i*_*,C*_*ji*_) and *ρ*(*C*_*i*_*,C*_*pi*_) represent the distances from point *C*_*i*_ to the classical domain *C*_*ji*_ and the junction domain *C*_*pi*_ respectively.

(4) Calculation of the comprehensive correlation degree


$$\begin{array}{c} K_{j}(S)=\begin{array}{c} n\\ \Sigma \\ i=\text{1} \end{array}W_{ki}\times K_{j}\left(C_{i}\right) \end{array}$$ (8)

where *K*_*j*_(*S*) represents the comprehensive correlation degree, *W*_*ki*_ represents the weight corresponding to *X*_*i*_, and max{*K*_*j*_ (*S*)} belongs to the level *j*, which represents the heavy metal ecological risk level of the evaluated object *S*.

To simultaneously account for both pollutant enrichment and biological toxicity, a dual correction of the integrated weights was performed in this study. First, the conventional exceedance weight (*W*_*ki*_) was calculated based on Equation (9), reflecting the average degree of exceedance for the *i-th* element relative to regional background values or assessment standards. Second, to address the limitations of traditional methods that overlook toxicological differences among elements, we incorporated the toxic response factor (*T*_r_^*i*^) proposed by Hakanson. The values assigned are as follows: Zn = 1, Ba = 2, Cu = Pb = 5, As=10, Ag = Mo = 15, and Sb = Hg = 40 [35,36]. This modification enhances the accuracy of our assessment of toxicological impacts. As illustrated in Equation (10), the modified weight (*W*_*ki*_^’^) is derived from the normalization of the product of the exceedance weight and the toxic response coefficient, representing the contribution of element *i* to the comprehensive risk. Finally, the comprehensive correlation degree (*K*_*j*_(*S*)), which indicates the affiliation of the evaluation object with risk grade j, was determined through the weighted summation of the modified weight (*W*_*ki*_^’^) and the single-factor correlation degree (*K*_*j*_(*C*_*i*_)) as per Equation (8). This improved calculation framework enhances the model’s sensitivity toward potentially toxic elements characterized by high toxicity and frequent exceedance, thereby aligning risk identification more closely with actual environmental scenarios.

Exceedance Multiplicity Weighting Method:


$$\begin{array}{c} W_{ki}=\frac{\frac{{(C}_{ki}}{\begin{array}{c} -\\ \Psi_{i} \end{array}})}{\left(\begin{array}{c} n\\ \Sigma \\ i=\text{1} \end{array}\frac{C_{ki}}{\begin{array}{c} -\\ \Psi_{i} \end{array}}\right)} \end{array}$$ (9)

Adjust the weight coefficient:


$$\begin{array}{c} \overset{\prime }{\underset{ki}{W}}=\frac{\left(W_{ki}\times \overset{i}{\underset{\text{r}}{T}}\right)}{\left(\begin{array}{c} n\\ \Sigma \\ i=\text{1} \end{array}W_{ki}\times \overset{i}{\underset{\text{r}}{T}}\right)} \end{array}$$ (10)

where $\begin{array}{c} -\\ \Psi_{i} \end{array}$ represents the arithmetic average of the evaluation grades, *W*_*ki*_ is the conventional weight coefficient calculated by the over-limit multiple weighting method, *W*_*ki*_^’^ is the modified weight coefficient, and *T*_r_^*i*^ is the toxicity response coefficient.

(5) Evaluation criterion

This study develops ecological risk assessment standards for potential toxic elements in soil by comprehensively referencing the soil environmental background values in Henan Province [37] and the national risk control standards for agricultural and development land [38,39]. Given that the farmland soil in the research area typically exhibits neutral to alkaline characteristics (pH > 7.5), the risk screening values and control values specified in the national standards for pH > 7.5 are uniformly adopted as evaluation criteria. For elements not addressed by national standards, such as Ag, Mo, Sb, and Ba, their threshold values are derived from relevant studies on soil evaluation in typical mining areas in China [40,41]. Based on varying risk levels, the assessment standards were categorized into five risk grades: clean, still clean, mild risk, moderate risk, and severe risk (Table 1). The determination of threshold values for each ecological risk grade adhered to the following principles: the threshold value for grade I was established at the upper limit of the regional soil background value; the threshold value for grade II was set at 30% of the soil environmental quality risk control standard; the threshold value for grade III was defined as 70% of the control standard; the threshold value for grade IV was directly aligned with the standard limit; and the threshold value for grade V was determined as 1.3 times the control standard value and above.

**Table 1:** Evaluation standard of soil heavy metal pollution.

Risk level	*ω* (mg·kg^-1^) of risk factors								
	Ag	Cu	Pb	Zn	Mo	Sb	Ba	As	Hg
Clean I	0.0700	22.0	23.6	61.5	0.570	0.900	502	10.0	0.0250
Still Clean II	87.6	120	150	150	140	12.0	1670	12.0	0.450
Low Risk III	204	280	350	350	326	28.0	3890	28.0	1.05
Moderate Risk IV	292	400	500	500	465	40.0	5560	40.0	1.50
Severe Risk V	380	520	650	650	605	52.0	7730	52.0	1.95

### 2.4. Positive matrix factorization (PMF) model

This paper uses the PMF 5.0 model developed by the US Environmental Protection Agency (EPA) to conduct source apportionment of potentially toxic elements in the farmland soil around the Baiyun Mountain mining area in Nanyang, China [42,43]. This technique falls under the category of receptor models, and it achieves risk source discrimination and contribution rate calculation by analyzing the chemical composition characteristics of environmental medium samples [44,45]. Compared with traditional source apportionment methods, the PMF model optimally utilizes the error analysis of measurement data points and imposes non-negative constraints on the factor matrix decomposition during the solution process, thus obtaining more practical physical significance in the analysis results [46–48]. The basic principle of source apportionment by the PMF model is as follows: Assuming that the sample concentration data matrix ***S***_*ik*_ can be decomposed into the factor score matrix $A_{ij}$, the factor loading matrix $B_{jk}$, and the residual matrix ***δ***_*ik*_, the basic equation is as follows:


$$\begin{array}{c} S_{ik}={\begin{array}{c} p\\ \Sigma \\ j=\text{1} \end{array}A}_{ij}\times B_{jk}+\delta_{ik}\;(\text{i}=\text{1},\text{2},\ldots ,\text{m};\text{k}=\text{1},\text{2},\ldots ,\text{n}) \end{array}$$ (11)

where ***S***_*ik*_ represents the concentration of the kth risk factor at the ith sample point; ***A***_*ij*_ represents the contribution of the ith sample point to the jth source; ***B***_*jk*_ represents the contribution concentration of the kth risk factor in the jth source; ***δ***_*ik*_ represents the residual matrix.

This model decomposes the original data matrix through multiple iterations using the weighted least squares method. Under the condition of non-negative constraints, the optimal matrices *A* and *B* are determined through the optimization process, and ultimately, *Q* reaches its minimum value:


$$\begin{array}{c} Q=\begin{array}{cc} n & m\\ \Sigma & \Sigma \\ i=\text{1} & k=\text{1} \end{array}{\left(\frac{S_{ik}-{\begin{array}{c} p\\ \Sigma \\ j=\text{1} \end{array}A}_{ij}\times B_{jk}}{\mu_{ik}}\right)}^{\text{2}}=\begin{array}{cc} n & m\\ \Sigma & \Sigma \\ i=\text{1} & k=\text{1} \end{array}{\left(\frac{\delta_{ik}}{\mu_{ik}}\right)}^{\text{2}} \end{array}$$ (12)

where *Q* represents the cumulative residuals, and *μ*_*ik*_ represents the uncertainty of the concentration of the kth risk factor at the ith sample point. Its formula is as follows:


$$\begin{array}{c} \mu_{ik}=\text{0}.\text{1}S_{ik}+MDL/\text{3} \end{array}$$ (13)

where *MDL* represents the detection limit value of each substance, and ***S***_*ik*_ represents the concentration of potentially toxic elements in the soil sample [49].

### 2.5. Statistical analysis

This study employed the ordinary kriging spatial interpolation technique using ArcGIS 10.4 software to visually analyze the spatial distribution characteristics of soil heavy metal content, ecological risk levels, and source factor contributions in the study area. Furthermore, to validate the objectivity and reliability of the evaluation results obtained from the improved matter element extension model, the Nemerow index method and the Muller index method were introduced for comparative analysis. The Nemerow Index emphasizes the impact of high-concentration pollutants on environmental quality, while the Geological Accumulation Index (*I*_*geo*_) quantitatively assesses the contribution of human activities to the enrichment of individual metals by comparing existing concentrations with geochemical background values. Through a comprehensive comparison of multiple evaluation methods, this study aims to provide a more thorough understanding of the ecological risk status of heavy metals in farmland soils within mining areas.

## 3. Results and discussions

### 3.1. Statistical characteristics of heavy metal content

**Fig 2:**
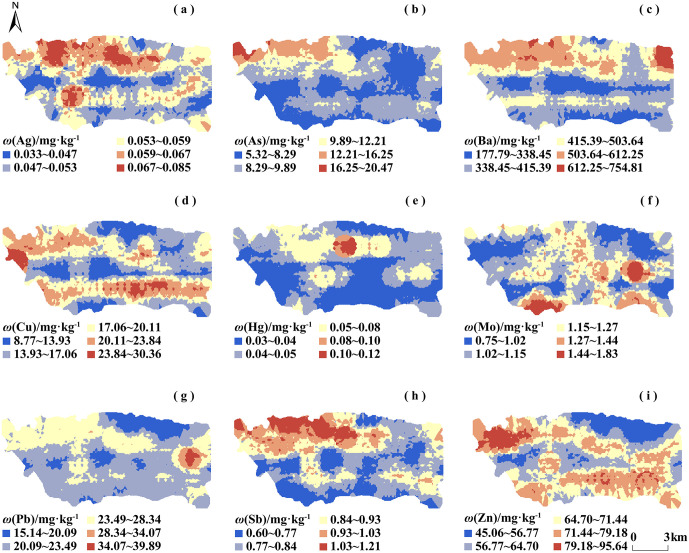
Spatial distribution of soil heavy metal content.

The analysis of heavy metal content in soil reveals varying degrees of enrichment for three elements: Zn, Mo, and Hg. Their average concentrations are 1.09, 2.02, and 1.84 times higher than the regional background values, respectively (Table 2), with sample proportions exceeding the standard being 40.50%, 38.67%, and 31.58%. Previous studies indicate that a coefficient of variation greater than 0.5 suggests an uneven spatial distribution of heavy metal content, indicating a risk of point source pollution potentially due to the introduction of exogenous substances [50]. In this study, the coefficients of variation for each element were as follows: Hg (0.80)> Cu (0.59)> As (0.52)> Ba (0.49)> Ag (0.43)> Mo (0.39)> Zn (0.34) ≈ Sb (0.34)> Pb (0.32). Notably, the coefficients of variation for Hg, Cu, and As exceed 0.5, suggesting possible pollution diffusion phenomena driven by anthropogenic factors such as agricultural activities and mineral extraction in the study area. Nine potential toxic elements exhibit significant spatial heterogeneity within the region. High-value areas are predominantly concentrated in the northwest mining area and its adjacent zones. Each element displays distinct distribution characteristics: Mo shows notable patchy enrichment in the central region, while Cu, Mo, and Zn demonstrate localized high-value aggregation in the southwestern region (Fig 2). From a spatial perspective, As, Ba, Hg, Pb, and Mo present an overall ‘island-like’ or ‘patchy’ distribution, characterized by prominent high-value centers and typical point source pollution traits. In contrast, Cu, Ag, Sb, and Zn exhibit a ‘strip-like’ or ‘continuous’ distribution, with their high-value concentrations being relatively dispersed. Based on the land use and sampling point layout illustrated in Fig 1, sampling points surrounding the northwest mining area show significantly elevated levels of Cu, Ag, Sb, and Zn compared to farmland areas located farther from the mining site, thereby directly confirming the substantial impact of mining activities on the adjacent soil environment. The localized high-value points identified in the central and southwestern regions may be attributed to concentrated agricultural practices, emissions from small-scale workshops, or elemental enrichment in the micro-terrain environments of these areas.

**Table 2:** Descriptive statistics of heavy metal content (n = 437).

Project	Ag	Cu	Pb	Zn	Mo	Sb	Ba	As	Hg
Background value (mg·kg^-1^)	0.0700	22.0	23.6	61.5	0.570	0.900	502	10.0	0.0250
Minimum detection limit (mg·kg^-1^)	0.0300	0.700	1.00	5.00	0.100	0.300	1.00	0.200	0.00200
Average value (mg·kg^-1^)	0.0550	17.5	22.7	66.8	1.15	0.850	426	9.25	0.0500
Standard deviation(mg·kg^-1^)	0.0240	10.4	7.31	22.5	0.440	0.280	208	4.78	0.0400
Maximum value (mg·kg^-1^)	0.163	126	72.6	175	3.42	2.30	1900	49.0	0.530
Minimum value (mg·kg^-1^)	0.0200	5.55	9.40	29.6	0.380	0.320	97.3	2.51	0.0100
Coefficient of variation	0.431	0.590	0.320	0.337	0.390	0.340	0.487	0.517	0.800

### 3.2. Ecological risk assessment of potentially toxic elements in soil

#### 3.2.1. The evaluation results of the matter-element extension analysis method.

This study analyzed and determined the heavy metal content of 437 surface soil samples. Now, the sampling point S_1_ is taken as a case for analysis. The test results of this sample site showed that the contents of each heavy metal (Ag, Cu, Pb, Zn, Mo, Sb, Ba, As and Hg) were 0.042, 9.667, 17.18, 50.86, 1.034, 0.677, 597.4, 6.646 and 0.038 mg·kg^-1^, respectively.

(1) Determine the matter-element matrix of S_1_ as:


$$\begin{array}{c} R_{\text{1}}=(\begin{array}{cc} \begin{array}{c} S_{\text{1}} \end{array} & \begin{array}{cc} \text{Ag} & \text{0}.\text{042}\\ \text{Cu} & \text{9}.\text{667}\\ \text{Pb} & \text{17}.\text{18} \end{array}\\ & \begin{array}{cc} \begin{array}{c} \text{Zn}\\ \text{Mo}\\ \begin{array}{c} \text{Sb}\\ \text{Ba}\\ \begin{array}{c} \text{As}\\ \text{Hg} \end{array} \end{array} \end{array} & \begin{array}{c} \text{5}\text{0}.\text{8}\text{6}\\ \text{1}.\text{0}\text{3}\text{4}\\ \begin{array}{c} \text{0}.\text{6}\text{7}\text{7}\\ \text{5}\text{9}\text{7}.\text{4}\\ \begin{array}{c} \text{6}.\text{6}\text{4}\text{6}\\ \text{0}.\text{0}\text{3}\text{8} \end{array} \end{array} \end{array} \end{array} \end{array}) \end{array}$$

(2) Establish the classical domain matrices *R* (1), *R* (2), *R* (3), *R* (4), *R* (5) and the segment domain matrix *R* (*p*) based on Table 1:


$$\begin{array}{c} R(\text{1})=(\begin{array}{ccc} N_{\text{1}} & \text{Ag} & \left(\text{0},\;\text{0}.\text{0}\text{7}\right)\\ & \text{Cu} & \left(\text{0},\;\text{2}\text{2}\right)\\ & \begin{array}{c} \text{Pb}\\ \text{Zn}\\ \begin{array}{c} \text{Mo}\\ \text{Sb}\\ \begin{array}{c} \text{Ba}\\ \text{As}\\ \text{Hg} \end{array} \end{array} \end{array} & \begin{array}{c} \left(\text{0},\;\text{2}\text{3}.\text{6}\right)\\ \left(\text{0},\;\text{6}\text{1}.\text{5}\right)\\ \begin{array}{c} \left(\text{0},\;\text{0}.\text{5}\text{7}\right)\\ \left(\text{0},\;\text{0}.\text{9}\right)\\ \begin{array}{c} \left(\text{0},\;\text{5}\text{0}\text{2}\right)\\ \left(\text{0},\;\text{1}\text{0}\right)\\ \left(\text{0},\;\text{0}.\text{0}\text{2}\text{5}\right) \end{array} \end{array} \end{array} \end{array})\;R(\text{2})=(\begin{array}{ccc} N_{\text{2}} & \text{Ag} & \left(\text{0}.\text{0}\text{7},\;\text{8}\text{7}.\text{6}\right)\\ & \text{Cu} & \left(\text{2}\text{2},\;\text{1}\text{2}\text{0}\right)\\ & \begin{array}{c} \text{Pb}\\ \text{Zn}\\ \begin{array}{c} \text{Mo}\\ \text{Sb}\\ \begin{array}{c} \text{Ba}\\ \text{As}\\ \text{Hg} \end{array} \end{array} \end{array} & \begin{array}{c} \left(\text{2}\text{3}.\text{6},\;\text{1}\text{5}\text{0}\right)\\ \left(\text{6}\text{1}.\text{5},\;\text{1}\text{5}\text{0}\right)\\ \begin{array}{c} \left(\text{0}.\text{5}\text{7},\;\text{1}\text{3}\text{9}.\text{5}\right)\\ \left(\text{0}.\text{9},\;\text{1}\text{2}\right)\\ \begin{array}{c} \left(\text{5}\text{0}\text{2},\;\text{1}\text{6}\text{6}\text{8}\right)\\ \left(\text{1}\text{0},\;\text{1}\text{2}\right)\\ \left(\text{0}.\text{0}\text{2}\text{5},\;\text{0}.\text{4}\text{5}\right) \end{array} \end{array} \end{array} \end{array}) \end{array}$$

$$\begin{array}{c} R(\text{3})=(\begin{array}{ccc} N_{\text{3}} & \text{Ag} & \left(\text{8}\text{7}.\text{6},\;\text{2}\text{0}\text{4}.\text{4}\right)\\ & \text{Cu} & \left(\text{1}\text{2}\text{0},\;\text{2}\text{8}\text{0}\right)\\ & \begin{array}{c} \text{Pb}\\ \text{Zn}\\ \begin{array}{c} \text{Mo}\\ \text{Sb}\\ \begin{array}{c} \text{Ba}\\ \text{As}\\ \text{Hg} \end{array} \end{array} \end{array} & \begin{array}{c} \left(\text{1}\text{5}\text{0},\;\text{3}\text{5}\text{0}\right)\\ \left(\text{1}\text{5}\text{0},\;\text{3}\text{5}\text{0}\right)\\ \begin{array}{c} \left(\text{1}\text{3}\text{9}.\text{5},\;\text{3}\text{2}\text{5}.\text{5}\right)\\ \left(\text{1}\text{2},\;\text{2}\text{8}\right)\\ \begin{array}{c} \left(\text{1}\text{6}\text{6}\text{8},\;\text{3}\text{8}\text{9}\text{2}\right)\\ \left(\text{1}\text{2},\;\text{2}\text{8}\right)\\ \left(\text{0}.\text{4}\text{5},\;\text{1}.\text{0}\text{5}\right) \end{array} \end{array} \end{array} \end{array})\;R(\text{4})=(\begin{array}{ccc} N_{\text{4}} & \text{Ag} & \left(\text{2}\text{0}\text{4}.\text{4},\;\text{2}\text{9}\text{2}\right)\\ & \text{Cu} & \left(\text{2}\text{8}\text{0},\;\text{4}\text{0}\text{0}\right)\\ & \begin{array}{c} \text{Pb}\\ \text{Zn}\\ \begin{array}{c} \text{Mo}\\ \text{Sb}\\ \begin{array}{c} \text{Ba}\\ \text{As}\\ \text{Hg} \end{array} \end{array} \end{array} & \begin{array}{c} \left(\text{3}\text{5}\text{0},\;\text{5}\text{0}\text{0}\right)\\ \left(\text{3}\text{5}\text{0},\;\text{5}\text{0}\text{0}\right)\\ \begin{array}{c} \left(\text{3}\text{2}\text{5}.\text{5},\;\text{4}\text{6}\text{5}\right)\\ \left(\text{2}\text{8},\;\text{4}\text{0}\right)\\ \begin{array}{c} \left(\text{3}\text{8}\text{9}\text{2},\;\text{5}\text{5}\text{6}\text{0}\right)\\ \left(\text{2}\text{8},\;\text{4}\text{0}\right)\\ \left(\text{1}.\text{0}\text{5},\;\text{1}.\text{5}\right) \end{array} \end{array} \end{array} \end{array}) \end{array}$$

$$\begin{array}{c} R(\text{5})=(\begin{array}{ccc} N_{\text{5}} & \text{Ag} & \left(\text{2}\text{9}\text{2},\;\text{3}\text{7}\text{9}.\text{6}\right)\\ & \text{Cu} & \left(\text{4}\text{0}\text{0},\;\text{5}\text{2}\text{0}\right)\\ & \begin{array}{c} \text{Pb}\\ \text{Zn}\\ \begin{array}{c} \text{Mo}\\ \text{Sb}\\ \begin{array}{c} \text{Ba}\\ \text{As}\\ \text{Hg} \end{array} \end{array} \end{array} & \begin{array}{c} \left(\text{5}\text{0}\text{0},\;\text{6}\text{5}\text{0}\right)\\ \left(\text{5}\text{0}\text{0},\;\text{6}\text{5}\text{0}\right)\\ \begin{array}{c} \left(\text{4}\text{6}\text{5},\;\text{6}\text{0}\text{4}.\text{5}\right)\\ \left(\text{4}\text{0},\;\text{5}\text{2}\right)\\ \begin{array}{c} \left(\text{5}\text{5}\text{6}\text{0},\;\text{7}\text{7}\text{2}\text{8}\right)\\ \left(\text{4}\text{0},\;\text{5}\text{2}\right)\\ \left(\text{1}.\text{5},\;\text{1}.\text{9}\text{5}\right) \end{array} \end{array} \end{array} \end{array})\;R(p)=(\begin{array}{ccc} N_{p} & \text{Ag} & \left(\text{0},\;\text{3}\text{7}\text{9}.\text{6}\right)\\ & \text{Cu} & \left(\text{0},\;\text{5}\text{2}\text{0}\right)\\ & \begin{array}{c} \text{Pb}\\ \text{Zn}\\ \begin{array}{c} \text{Mo}\\ \text{Sb}\\ \begin{array}{c} \text{Ba}\\ \text{As}\\ \text{Hg} \end{array} \end{array} \end{array} & \begin{array}{c} \left(\text{0},\;\text{6}\text{5}\text{0}\right)\\ \left(\text{0},\;\text{6}\text{5}\text{0}\right)\\ \begin{array}{c} \left(\text{0},\;\text{6}\text{0}\text{4}.\text{5}\right)\\ \left(\text{0},\;\text{5}\text{2}\right)\\ \begin{array}{c} \left(\text{0},\;\text{7}\text{7}\text{2}\text{8}\right)\\ \left(\text{0},\;\text{5}\text{2}\right)\\ \left(\text{0},\;\text{1}.\text{9}\text{5}\right) \end{array} \end{array} \end{array} \end{array}) \end{array}$$

(3) Correction of heavy metal weights

**Fig 3:**
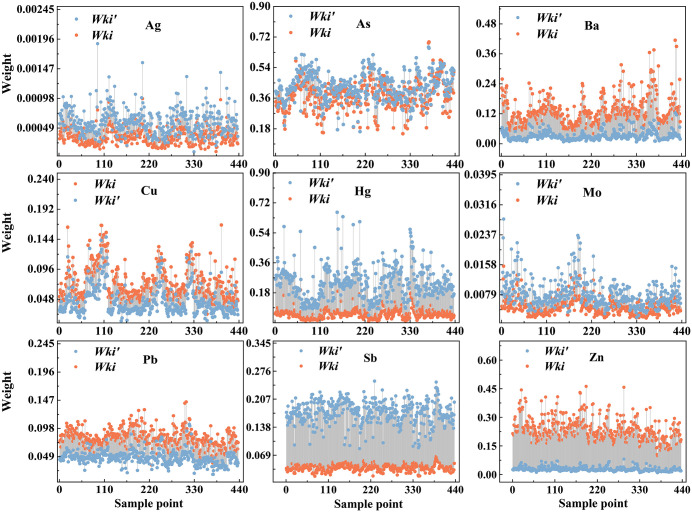
Comparative analysis of weight values of soil heavy in the study area.

The weight distribution of heavy metal elements was analyzed based on the over-limit multiple method and the modified weighting method (Fig 3). The results show that there are significant differences in the weight values of the two methods. Specifically, the corrected weight values (*W*_*ki*_*’*) of the four types of elements Ba, Cu, Pb, and Zn showed a significant decrease compared with the weight values (*W*_*ki*_) of the traditional method, and the decreases reached 76.09%, 40.04%, 42.24%, and 88.16% respectively. The corrected weight values of the five heavy metal elements, namely Ag, As, Hg, Mo and Sb, have significantly increased. Among these elements, As exhibited the smallest increase (13.54%), followed by Mo, Ag, and Hg with increases of 25.67%, 78.92%, and 132.45%, respectively. Notably, Sb showed the most substantial growth, reaching a significant increase of 353.54%. The revised comprehensive weight Coefficient (*W*_*ki*_) can not only reflect the ecotoxicity of each element, but its numerical change also characterizes the environmental accumulation characteristics of potentially toxic elements. Compared with the conventional weighting method that only considers the concentration difference of potentially toxic elements, the improved algorithm shows more significant parameter sensitivity and environmental adaptability in ecological risk assessment.

(4) Evaluation results

**Fig 4:**
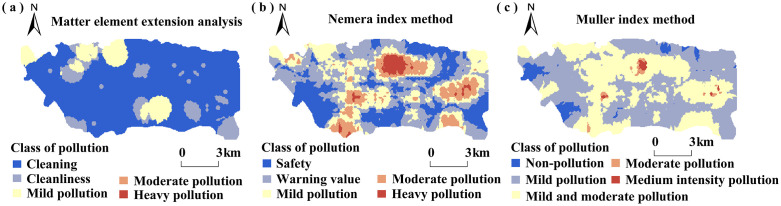
Spatial distribution of heavy metal pollution grade evaluation of topsoil.

Based on formulas (4) to (7), the correlation degree of 437 soil heavy metal samples was calculated to obtain the correlation degree *K*_*j*_ (*φ*_*i*_) corresponding to each evaluation grade. The higher the value, the stronger the correlation between potentially toxic elements and this evaluation grade. Taking sampling point S1 as an example, its comprehensive correlation degree indicators are 0.209, 0.004, −0.674, −1.029 and −0.379 respectively. According to the ecological risk level discrimination criterion, the maximum comprehensive correlation degree value of this sampling point is max{*K*_*2*_(*N*)}= 0.209. This point is classified as level I, indicating that the soil in this area is in a relatively clean state. For this reason, 410 sites (accounting for 93.82%) in the study area presented grade I ecological risk, 24 sites (5.49%) reached grade II ecological risk, and 3 sites (0.69%) showed grade V ecological risk. On this basis, the Spatial Analyst tool of Geographic Information System (ArcGIS 10.4) was adopted, combined with the Kriging spatial interpolation method, to analyze the spatial distribution of heavy metal ecological risks in the study area (Fig 4a). The results show that the overall state of the study area is clean, the ecological risk of potentially toxic elements is relatively low, and the main risk areas occur in the northwest and the south-central regions.

#### 3.2.2. The evaluation results of the Nemerow comprehensive index method and the geological accumulation index method.

The evaluation results of the Nemerow comprehensive index method indicate that the regional *P*_*N*_ index ranges from 0.89 to 15.32, with an average value of 1.89, demonstrating significant spatial heterogeneity characteristics (Fig 4b). Among all sampling points, 9 monitoring points (accounting for 2.06%) exhibited ecological risk levels reaching the alert threshold of Grade II, while 296 points (accounting for 67.73%) displayed mild risk levels classified as Grade III. Notably, mercury emerged as the primary risk factor, ranking first in both the over-limit rate and risk contribution rate. The evaluation results of the Muller index method reveal that the ecological risks associated with each heavy metal, ranked from weak to strong, are as follows: Cu < Ag < Ba <As <Sb < Pb < Zn < Hg < Mo, with Mo presenting the highest risk level. The spatial distribution of heavy metal risk levels illustrated in Fig 4c shows that approximately 65.68% of the area is categorized as having a mild risk level of Grade II, 18.31% of the area falls within a light to moderate-intensity risk level of Grade III or above, and only 16.02% of the area is classified as risk-free (Grade I). Areas with a higher degree of risk are primarily concentrated in the central, southwestern, and southeastern regions of the study area. This concentration may be attributed to the accumulation of potentially toxic elements in the soil as a result of mining activities in the region.

#### 3.2.3. Analysis of multi-model evaluation results.

A comprehensive comparison of the three evaluation methods reveals that the proportion of points classified as “clean” by the improved matter element extension model is significantly higher than that identified by the Nemerow comprehensive index method and the geological accumulation index method. This notable discrepancy in evaluation outcomes underscores the varying response mechanisms of different models to the complex geochemical backgrounds present in mining areas. The Nemerow index method, characterized by its reliance on weighted averages and extreme value amplification, significantly amplifies the influence of the maximum pollution factor. Consequently, the evaluation results are susceptible to local extreme values, often leading to extensive areas being categorized as “mild risk.” In contrast, the Muller index fails to account for the toxicological differences among heavy metals and exhibits a low tolerance for natural enrichment backgrounds, resulting in a tendency to overestimate risks in regions characterized by natural mineralization. In contrast, the improved matter element extension model enhances the scientific rigor of evaluation through a dual mechanism of ‘toxicity weighting’ and ‘fuzzy discrimination’. For instance, although elements such as Zn and Mo are enriched to varying degrees, their contributions to the overall risk are diminished through weight optimization due to their low biological toxicity. The model demonstrates heightened sensitivity to highly toxic elements such as Hg and Pb. Additionally, the matter element model quantifies the degree of belonging of samples to various risk levels using a continuous correlation function, effectively avoiding the ‘abrupt change’ in evaluation results that can occur due to small numerical differences at the classification threshold in traditional models. Consequently, the results of the matter element model not only accurately identify the real risk points surrounding the northwest mining area but also reasonably assess the cleanliness status of remote farmland, making its evaluation results more objective and targeted.

### 3.3. Correlation analysis of potentially toxic elements in soil

**Fig 5:**
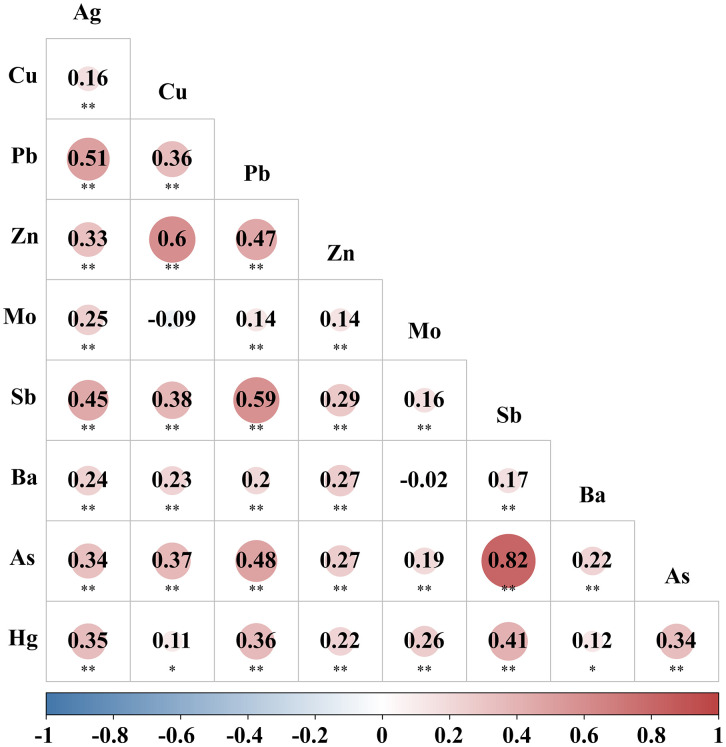
Analysis results of soil heavy metal correlation. * p < 0.05; ** p < 0.01.

The correlation among heavy metal elements serves as a crucial indicator for identifying the sources of pollutants. A significant positive correlation among these elements typically signifies that they share homologous input characteristics. Studies have shown that the greater the correlation coefficient among potentially toxic elements in soil, the greater the possibility that they come from the same pollution source; otherwise, there may be multiple pollution sources [51]. It can be known from Fig 5 that the four groups of elements Ag-Pb, Cu-Zn, Pb-Sb and Sb-As all show significant positive correlations (R > 0.5, p < 0.01), indicating that these heavy metal elements may have homologous pollution or combined pollution. However, Ba and Mo elements did not show significant correlations with other (p > 0.05), suggesting that the two might have independent pollution sources.

### 3.4. Analysis of sources of potentially toxic elements in soil

To further enhance the effectiveness of prevention and control measures in high-risk areas, specifically the northwest and central-south regions, it is essential to clarify the pollution sources that contribute to these risk hotspots and quantify their respective contributions. Therefore, this study employs the PMF model for the quantitative source apportionment of heavy metals, aiming to accurately identify the targeted sources and the characteristics of their contributions to high-risk factors. This will provide a scientific basis for formulating tailored farmland management policies in mining areas.

To improve the accuracy of source apportionment of potentially toxic elements in soil, a comparative experiment of multi-scenario simulation was adopted. Three to six potential factors were set successively to systematically evaluate the influence of different factor numbers on the apportionment results. Relevant studies suggest that the optimal number of factors is attained when the parameter (*Q*_*robust*_/*Q*_*expected*_) demonstrates the maximum reduction [52]. The experimental results of this study show that when the number of factors increases from 4 to 5, *Q*_*robust*_/*Q*_*expected*_ decreases significantly from 2.34 to 1.63, with the greatest decrease. To ensure the accuracy of source apportionment, this study incorporated nine heavy metals into the PMF 5.0 model. Based on the signal-to-noise ratio (S/N) and data quality, silver (Ag) was classified as a weak variable, while the remaining eight elements were designated as strong variables. The fitting results, which compare the predicted values of the model with the observed values, are presented in Table 3. With the exception of Ag, the R² values for all other elements exceed 0.5, indicating that the PMF model effectively captures the distribution characteristics of heavy metals in the soil of the study area.

**Table 3:** Fitting results of measured value and simulated value of soil heavy metal content.

Heavy metal	*R* ^2^	Slope	Intercept
Ag	0.373	0.333	0.030
Cu	0.721	0.651	5.450
Pb	0.508	0.547	9.330
Zn	0.664	0.645	21.140
Mo	0.761	0.661	0.350
Sb	0.803	0.907	0.060
Ba	0.998	0.979	8.320
As	0.950	0.842	1.350
Hg	0.947	0.812	0.010

**Fig 6:**
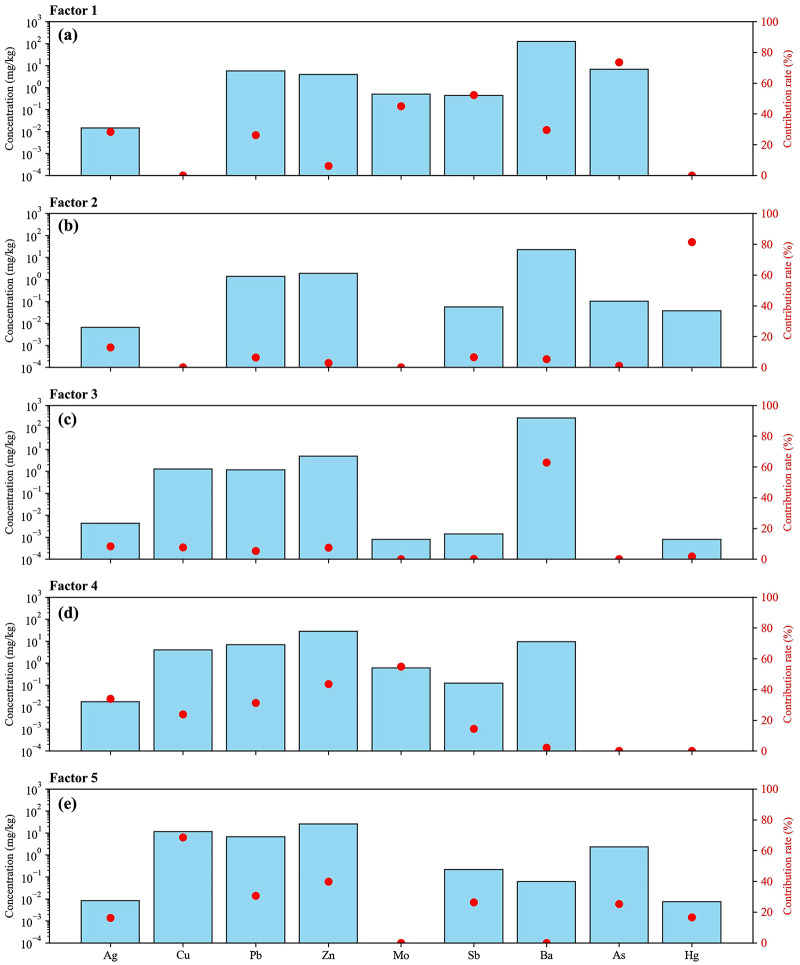
Factor profiles and contribution rates of each potentially toxic element based on the PMF model. Blue bars represent the concentrations of potentially toxic elements, and red dots represent the contribution percentage of each factor to the total concentration of each element.

**Fig 7:**
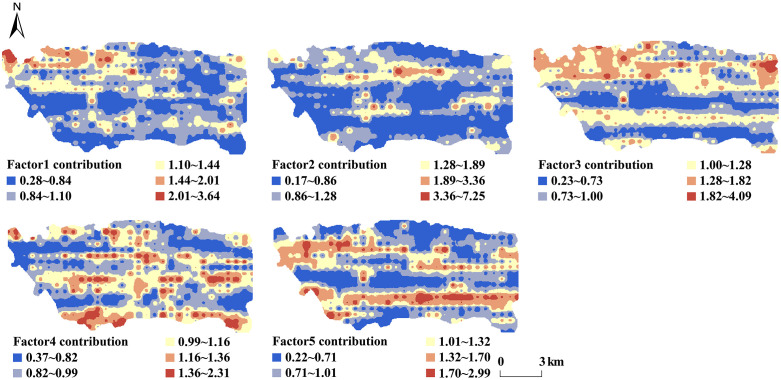
Spatial distribution of contribution for each soil potentially toxic elements source factor. All map layers were created by the authors. Administrative boundaries were obtained from the National Catalogue Service for Geographic Information of China (http://www.webmap.cn/). The visualization of evaluation results were digitized based on the authors’ field survey data and model simulation results. No copyrighted basemaps were used in the production of these figures.

Fig 6 illustrates the contribution rates of five pollution sources to each heavy metal. Among these, factor 1 exhibits the highest contribution to As and Sb, accounting for 73.7% and 52.3%, respectively. According to Table 2, the average content of As in the study area is 9.25 mg·kg^-1^, which is close to the background value of the soil environment in Henan Province (10 mg·kg^-1^), China. This suggests that the arsenic levels may be influenced by natural parent materials [53]. The spatial distribution displayed in Fig 7a demonstrates a strong spatial consistency between the high-value areas of As, factor 1, and the valley zone. This consistency arises from the fact that river and lake sediments in the valley area typically possess a fine mechanical composition, such as clay components, which exhibit significant adsorption and enrichment effects on As. Furthermore, the reduction in terrain slope facilitates the migration of heavy metals along surface runoff to the low-lying areas of river valleys, leading to secondary accumulation [54]. Concurrently, the data in Table 2 indicate that the coefficient of variation (CV = 0.34) for Sb is considerably lower than that of other potentially toxic elements, and its spatial distribution reflects typical characteristics of natural sources [55]. Specifically, the average Sb content in the soil of the study area (0.85 mg·kg^-1^) is 5.6% lower than the regional background value of 0.9 mg·kg^-1^. Furthermore, considering its occurrence form in the crust, which is primarily derived from the weathering and erosion of Sb-containing minerals, it exhibits characteristics typical of non-human interference. Therefore, Factor 1 can be interpreted as a natural parent material source.

The primary loading element of Factor 2 is Hg, which accounts for a contribution rate of 81.5%. The data presented in Table 2 reveals that the coefficient of variation for the Hg element is as high as 0.8 (CV = 0.8), significantly exceeding that of other heavy metal elements. This suggests a greater degree of interference from human activities [56]. Fig 7b illustrates that the contribution rate of this factor exhibits considerable variability across the region; however, notable high-value centers are observed in proximity to residential areas located in the central and northern parts. Previous studies have indicated that the abnormal enrichment of Hg in soil is predominantly influenced by atmospheric deposition [57,58]. Furthermore, based on standardized energy survey data, 67% of residents in the study area depend on coal for their daily energy consumption, particularly during the winter heating period (November to March), when gaseous Hg released from coal enters the surface system via atmospheric dry and wet deposition. Consequently, Factor 2 is interpreted as the atmospheric deposition source arising from fossil fuel combustion.

Factor 3 demonstrated a significant independent contribution of 62.9% to Ba. As an alkaline earth metal belonging to the IIA group, Ba’s outer electronic configuration facilitates the formation of stable ionic compounds with halogens and elements from the oxygen group in soil environments (e.g., BaCl_2_, Ba(NO_3_)_2_, Ba(OH)_2_). Existing studies indicate that the primary sources of Ba in natural soil are the weathering of natural parent materials and contributions from mining activities [59]. The results of this study indicate that the coefficient of variation for Ba is 0.487, signifying moderate spatial heterogeneity. This suggests that the distribution of its content may be influenced by specific human activities [60]. The spatial distribution characteristics (Fig 7c) illustrate that the high contribution rate of Ba is concentrated in the northwest and northeast edges of the study area, where local concentrations exceed the soil background value in Henan Province. In terms of regional background, non-metallic minerals such as limestone and shale are extensively distributed in the northwest and northeast of the research area, and the processing industries for building materials, including cement and bricks, are clustered in these regions [61]. Ba is commonly found as a companion component of these non-metallic minerals or may enter the soil through dust emissions during the production processes of building materials. The absence of significant correlations between Ba and metal minerals such as Cu, Pb, and Zn further underscores its unique source [62]. Therefore, Factor 3 is identified as a source from the non-metallic mining and building materials industry.

Factor 4 exhibits significant loadings on potentially toxic elements such as Mo, Ag, Zn and Pb, with contribution rates of 54.9%, 43.6%, 31.4%, and 33.9% respectively. The high load characteristics of Mo and Ag are closely aligned with the geological context of the Baiyun Mountain mining area [63]. This region is situated within the Funiu Mountain polymetallic mineralization belt, primarily characterized by porphyry skarn-type molybdenum deposits, which are frequently associated with elements such as Pb, Zn, Ag [63]. The average concentration of Mo in the study area is 1.15 mg·kg^-1^, which is approximately twice the background value, and the spatial distribution reveals a strong coupling between high-value areas and the locations of mineral deposits. This suggests that dust diffusion during mining operations, mechanical crushing, and the long-term accumulation of tailings are the primary pathways for the input of Mo and Ag [64]. Meanwhile, Pb and Zn are recognized as indicator elements of traffic sources, primarily originating from motor vehicle exhaust emissions as well as tire and brake pad wear [4]. Although these elements may arise from various emission activities, the spatial distribution characteristics (Fig 7d) reveal a distinct strip-shaped feature for the contribution rate of factor 4, which closely aligns with the direction of the main transportation arteries in the study area. Due to the highly consistent spatial diffusion pathways of pollution sources, significant superposition effects often occur in the ecosystems of mining areas. Existing source apportionment studies employing PMF models in these ecosystems demonstrate that ore spillage during logistics activities, the dispersion of ore-containing dust (Mo, Ag), and exhaust emissions from heavy transport vehicles (Pb, Zn) have undergone profound coupling at receptor sites [65,66]. However, constrained by the matrix factorization principles based on data variance in the PMF model, the highly coordinated spatiotemporal distribution of composite pollution is often challenging to analyze independently using mathematical models. Consequently, factor 4 is interpreted as a mixed source arising from mining development and transportation logistics.

Among factor 5, Cu exhibits the strongest load at 68.5%, followed by Zn at 39.8%. The correlation coefficient of 0.6 indicates significant homology. Spatial analysis (Fig 7e) reveals that the contribution rate of this factor demonstrates a large-scale continuous high-value distribution in the mining activity areas and surrounding farmland in the southern and central parts of the study area, reflecting a collaborative pollution process under the composite background of “mining agriculture [67].” Cu is primarily a core component of fungicides, such as copper sulfate (CuSO_4_), while Zn serves as an essential trace element fertilizer for crop growth [68]. Field investigations have confirmed that fertilizers, particularly phosphate and organic fertilizers used on economic crops like rice and peanuts in the study area, often contain elevated levels of Zn as an additive or accompanying impurity. Long-term agricultural inputs contribute to the synchronous accumulation of Cu and Zn in the surface soil of farmland [69]. Furthermore, Cu-containing tailings generated by mining operations are prone to geochemical migration and diffusion to low-lying farmland during the stacking process due to rainwater leaching and surface runoff. This complex pollution pattern, characterized by overlapping spatial locations of receptors, is highly typical in the multi-metal mining – farmland complex ecosystem. Recent literature on PMF source apportionment based on spatial distribution has confirmed that when agricultural non-point source inputs significantly overlap with the runoff leaching pathways of upstream mining tailings, PMF models often identify them as mixed factors exhibiting strong synergistic cumulative effects due to the similar hydrological driving mechanisms of pollutants [70,71]. The combination of leaching emissions from mining activities and long-term agricultural inputs is the primary cause of heavy metal exceedance in these areas. Consequently, factor 5 is identified as a mixed source of pesticide use and mining leaching emissions.

In summary, there are five primary sources of heavy metal pollution in the farmland surrounding the Baiyun Mountain mining area in Nanyang, China. These include natural parent material sources (26.1%), mixed sources from transportation and mining development (25.8%), mixed sources resulting from pesticide use and mining emissions (19.8%), atmospheric deposition from fossil fuel combustion (15.1%), and sources from non-metallic mineral mining and building material industry (13.2%). The results indicate that the composite effects of ‘mining, transportation, and agriculture,’ represented by factors 4 and 5, are the primary driving force behind the accumulation of heavy metals in the soil of the study area, contributing a cumulative total of 45.6%. Further analysis indicates that the serious risk point (Grade V) in the northwest of the study area significantly overlaps with the high contribution area of Factor 4 (transportation and mining development). In contrast, the mild risk areas in the central and southern regions are primarily driven by Factor 5 (pesticide use and mining development). This confirms that dispersion from transportation activities and tailings leaching associated with mining, as well as atmospheric deposition from fossil fuel combustion, are the primary external inputs driving the increase in ecological risks in the study area. Accordingly, future farmland protection policies should strengthen the prevention and control of tailings leaching and traffic-generated dust, while also addressing the sources of atmospheric deposition in a synergistic manner. Optimizing the regional energy structure will allow for the precise control of heavy metal pollution. By focusing on mitigating these high-risk sources, it is anticipated that maximum ecological risk restoration can be achieved at the lowest governance cost.

## 4. Conclusions

This study has certain limitations. Due to objective constraints, the simultaneous collection of undisturbed local control soil samples may result in limited resolution when distinguishing between natural geochemical backgrounds and minor mining pollution. Although this study utilized the soil environmental background values in Henan Province as a regional reference and obtained relatively robust results in the PMF model, future research should strive to further refine the characterization of the natural input contribution of heavy metals in farmland surrounding the mining area by collecting deep soil samples or selecting control points located far from the mining area. Additionally, the enrichment and correlation of potential toxic elements in soil are influenced not only by pollution sources but also by soil physicochemical properties such as clay content, organic matter, and iron-manganese oxides. The lack of synchronous measurement of these key physical and chemical indicators in this study may cause the factors analyzed by PMF to couple the natural soil formation process with the adsorption characteristics of specific soil components, potentially leading to partial overestimation or underestimation of human contribution rates. Future research should incorporate soil physicochemical parameters as constraint variables in multidimensional model analysis to more accurately distinguish between human and natural contributions.

Based on an analysis of 437 soil samples collected from farmland surrounding the Baiyun Mountain mining area in Nanyang, China, this study examines the spatial differentiation characteristics of nine heavy metal elements, including Ag, Cu and Pb, etc. An ecological risk assessment model was developed utilizing matter element extension theory to evaluate the ecological risks posed by potentially toxic elements in the region. Concurrently, a positive definite matrix factorization (PMF) model was employed to analyze the sources of heavy metal pollutants. The results indicated that elements such as Ag, Cu, Pb, Zn, Mo, Sb, Ba, As, and Hg exhibited significant spatial heterogeneity, with average concentrations of 0.055, 17.5, 22.72, 66.84, 1.15, 0.85, 426.39, 9.25, and 0.05 mg·kg^-1^, respectively. The enrichment levels of Zn and Mo were significantly higher than the soil background values, forming a composite distribution pattern resembling an island block centered around the mining area. In contrast, the enrichment of Hg predominantly occurs in agricultural and residential areas. Its spatial distribution exhibits point-like enrichment characteristics associated with fossil fuel combustion and agricultural activities. The ecological risk assessment results reveal that 93.82% of the samples are classified as clean, 5.49% as slightly polluted, and 0.69% as severely polluted, with Hg identified as the primary pollutant factor. The study area primarily comprises five types of pollution sources: natural parent material sources (26.1%), mixed sources from transportation and mining development (25.8%), mixed sources resulting from pesticide use and mining emissions (19.8%), atmospheric deposition from fossil fuel combustion (15.1%), and sources from non-metallic mineral mining and building material industry (13.2%). Mining development, agricultural activities, and atmospheric deposition from fossil fuel combustion are the main controlling factors for heavy metal pollution in the region. This study offers valuable support for fundamental theoretical research on the restoration of degraded ecosystems in mining areas, as well as the development of response technologies.

## Supporting information

S1 Table The datasets used in this study. (XLSX)
